# Clinical and histological significance of urinary CD11c^+^ macrophages in lupus nephritis

**DOI:** 10.1186/s13075-020-02265-1

**Published:** 2020-07-17

**Authors:** Jihye Kim, Jung Sun Lee, Heounjeong Go, Joon Seo Lim, Ji Seon Oh, Yong-Gil Kim, Chang-Keun Lee, Bin Yoo, Seokchan Hong

**Affiliations:** 1 Asan Institute for Life Sciences, Asan Medical Center, Seoul, Republic of Korea; 2 Division of Rheumatology, Department of Internal Medicine, Asan Medical Center, University of Ulsan College of Medicine, Seoul, Republic of Korea; 3 Division of Rheumatology, Department of Internal Medicine, Seoul Veterans Hospital, Seoul, Republic of Korea; 4 Department of Pathology, Asan Medical Center, University of Ulsan College of Medicine, Seoul, Republic of Korea; 5 Clinical Research Center, Asan Medical Center, University of Ulsan College of Medicine, Seoul, Republic of Korea

**Keywords:** Lupus nephritis, Urine, Macrophage, Tubulointerstitial change

## Abstract

**Background:**

Infiltration of immune cells into the kidney is one of the key features of lupus nephritis (LN). The presence of immune cells in the urine may be used as a non-invasive biomarker of LN. Here, we aimed to analyze the clinicopathologic significance of urinary CD11c^+^ macrophages in patients with LN.

**Methods:**

The numbers and proportions of CD11c^+^ macrophages in the urine samples of patients with LN at the time of kidney biopsy were examined using flow cytometry. We also examined the association between the levels of urinary CD11c^+^ macrophages and the clinical and pathologic features of patients with LN.

**Results:**

Compared with patients without LN or those with non-proliferative LN, patients with proliferative LN had significantly higher numbers and proportions of urinary CD11c^+^ macrophages, which were strongly correlated with the serum anti-dsDNA antibody titer. The numbers and proportions of urinary CD11c^+^ macrophages were significantly associated with the values of chronicity indices such as tubular atrophy and interstitial fibrosis. No significant relationships were found between the levels of urinary CD11c^+^ macrophages and the activity scores, degree of proteinuria, or lupus disease activity. Urinary CD11c^+^ macrophages were more abundant in patients who did not achieve renal response to induction treatment with immunosuppressants than in those who achieved complete or partial response. The receiver operating characteristic (ROC) curve analysis showed that the number of urinary CD11c^+^ macrophages was the most powerful predictor of renal response at 6 months (ROC-AUC = 1.00, *p* = 0.0004).

**Conclusion:**

The urinary levels of CD11c^+^ macrophages were closely associated with the chronic pathologic changes of LN and renal response and may thus be used as a novel biomarker in LN.

## Background

Systemic lupus erythematosus (SLE) is a systemic autoimmune disease that commonly involves the kidney as a form of lupus nephritis (LN) and results in considerable morbidity and mortality [1]. LN is characterized by high titers of autoantibodies such as the anti-dsDNA antibody, and immune complex-mediated injury to the kidney [2, 3]. LN is initiated after deposition of the immune complex in the glomeruli; however, tubulointerstitial inflammation is commonly observed in LN. Accordingly, several studies suggested that tubulointerstitial damage is a potent prognostic factor for long-term renal outcomes in LN [4, 5]. Nevertheless, little is known about the specific types of cells and mechanisms that are involved in the tubulointerstitial changes in LN.

Kidney biopsy is essential for obtaining accurate diagnosis and classification of patients with LN. However, considering the invasive nature of tissue biopsy, attempts have been made to develop alternative non-invasive methods for LN diagnosis and classification [6, 7]. Several recent studies have detected urinary cells in patients with LN and showed that the kidney-originated cells in the urine of patients with LN had strong correlations with disease activity and may thus be used as new biomarkers [8–11].

We recently reported that CD11c^+^ cells comprised the majority of immune cells in the urine of patients with LN and that these cells had the phenotypes of macrophages and may be actively involved in the tubulointerstitial damages in LN [12]. Here, we analyzed the clinical and pathological significance of urinary CD11c^+^ macrophages in patients with LN.

## Patients and methods

### Study subjects and samples

All clinical samples used in this study were collected between June 2014 and December 2019 at Asan Medical Center, a tertiary referral hospital in Seoul, South Korea, from patients with LN who fulfilled the American College of Rheumatology classification criteria for SLE. LN was confirmed by kidney biopsy and classified in accordance with the International Society of Nephrology/Renal Pathologic Society (ISN/RPS) 2003 classification system [13]. Urine samples were collected early in the morning on the day of biopsy from 41 patients with active LN (proliferative and non-proliferative). Urine cell isolation was performed after urine collection and stored in liquid nitrogen for future flow cytometric analysis. We included 10 patients with inactive SLE without LN, 5 patients with active SLE without LN, 5 patients with inactive LN, and 6 patients with other kidney diseases (IgA nephropathy (*n* = 3) and ANCA-associated vasculitis (*n* = 3)) as control groups in the analysis. The characteristics of all the subjects are presented in Table 1. The protocols of this study were approved by the institutional review board of Asan Medical Center (IRB No. 2014-0568, Seoul, Korea). Written informed consent was obtained from all patients and control subjects prior to their inclusion in the study.

**Table 1:** Baseline characteristics of included patients with or without lupus nephritis

	Inactive SLE (*N* = 10)	Active SLE (*N* = 5)	Inactive LN (*N* = 5)	Active LN (*N* = 41)	*P* value
Age (years)	40.7 ± 14.5	30.8 ± 10.6	36.6 ± 4.7	35.0 ± 13.5	0.515
Female	10 (100)	5 (100)	5 (100)	36 (87.8)	0.136
Hypertension	4 (40)	0 (0)	1 (20)	18 (44)	0.369
Diabetes mellitus	0 (0)	0 (0)	0 (0)	1 (2.4)	0.524
Laboratory data					
Creatinine (mg/dL)	0.61 (0.56–0.72)	0.55 (0.39–0.69)	0.79 (0.60–1.08)	0.68 (0.57–0.88)	0.135
eGFR (ml/min/1.73 m^2^)	117 (102–126)	133 (115–135)	94 (72–117)	108 (81–128)	0.180
Albumin (g/dL)	3.6 (3.5–3.9)	3.8 (3.2–4.2)	3.7 (3.6–4.0)	2.7 (2.1–3.3)	< 0.001
C3^a^ (mg/dL)	69.1 ± 20.0 (*n* = 9)	71.0 ± 37.2	68.2 ± 15.9	52.0 ± 25.7	0.120
C4^a^ (mg/dL)	13.6 ± 6.5 (*n* = 9)	8.4 ± 6.2	17.6 ± 6.5	12.6 ± 10.1	0.460
Anti-dsDNA ab^a^ (IU/mL)	18.1 (9.4–30.0) (*n* = 9)	60.1 (20.1–89.2)	10.4 (6.9–30.8)	85.4 (24.4–297.0)	0.029
Proteinuria^a^ (mg/g)	147.8 (70.3–316.5) (*n* = 9)	179.1 (*n* = 3)	135.1 (96.9–349.1)	1715.1 (949.8–4429.9)	< 0.001
Urine specific gravity	1.017 ± 0.008	1.014 ± 0.007	1.017 ± 0.008	1.016 ± 0.006	0.811
Lupus involvement					
Kidney	0 (0)	0 (0)	5 (100)	41 (100)	< 0.001
Joint	0 (0)	5 (100)	0 (0)	5 (12.2)	0.437
Hematologic	2 (20)	4 (80)	3 (60)	16 (39)	0.748
Serositis	0 (0)	0 (0)	0 (0)	10 (24.4)	0.029
Constitutional	0 (0)	3 (60)	0 (0)	15 (36.6)	0.080
CNS	0 (0)	2 (40)	0 (0)	3 (7.3)	0.901
SLEDAI^a^	3.9 ± 1.3	14.6 ± 5.7	3.4 ± 0.9	14.1 ± 6.4	< 0.001
Lupus nephritis histologic class					< 0.001
II	0 (0)	0 (0)	0 (0)	5 (12.2)	
III	0 (0)	0 (0)	3 (60)	13 (31.7)	
IV	0 (0)	0 (0)	2 (40)	10 (24.4)	
II + V	0 (0)	0 (0)	0 (0)	1 (2.4)	
III + V or IV + V	0 (0)	0 (0)	0 (0)	4 (9.8)	
V	0 (0)	0 (0)	0 (0)	8 (19.5)	
Treatment					
Hydroxychloroquine	9 (90)	5 (100)	5 (100)	40 (97.6)	0.348
Steroids					0.004
None	4 (40)	0 (0)	3 (60)	2 (4.9)	
Low dose	2 (20)	0 (0)	2 (40)	6 (14.6)	
Medium dose	4 (40)	3 (60)	0 (0)	17 (41.5)	
High dose	0 (0)	2 (40)	0 (0)	14 (34.1)	
Pulse	0 (0)	0 (0)	0 (0)	2 (4.9)	
Immunosuppressive drugs					
None	8 (80)	0 (0)	0 (0)	4 (9.8)	< 0.001
Intravenous cyclophosphamide	0 (0)	1 (20)	2 (40)	13 (31.7)	0.052
Mycophenolate mofetil	2 (20)	1 (20)	5 (100)	22 (53.7)	0.045
Tacrolimus	0 (0)	1 (20)	2 (40)	19 (46.3)	0.005
Azathioprine	0 (0)	1 (20)	1 (20)	7 (17.1)	0.260
Methotrexate	0 (0)	2 (40)	0 (0)	0 (0)	0.120

Results expressed as the mean ± standard deviation, median (interquartile range), or number (%)

*eGFR* estimated glomerular filtration rate

^a^Missing values were excluded from analysis

### Patient data collection

The SLE Disease Activity Index (SLEDAI), a measure of lupus disease activity, was assessed [14]. Inactive SLE patients were those with SLEDAI ≤ 6 without renal involvement at the time of enrollment. Inactive LN patients were those with a history of LN with complete renal response at the time of the study. Mixed proliferative and membranous histology (class III or IV with V) were considered as proliferative LN. Complete renal response was defined as a urine protein-to-creatinine ratio of < 500 mg/g and normal or near-normal glomerular filtration rate (estimated glomerular filtration rate (eGFR); within 10% of normal eGFR if previously abnormal) based on the Joint European League Against Rheumatism and European Renal Association-European Dialysis and Transplant Association recommendations [15]. Partial response was defined as ≥ 50% reduction of proteinuria and normal or near-normal eGFR. Patients who achieved complete or partial renal responses at 6 months after induction therapy were considered as having positive renal response.

### Flow cytometric analysis

To exclude dead cells from analysis, cells were incubated with Fixable Viability Dye eFluor 506 (eBioscience, San Diego, CA, USA) for 30 min at 4 °C in the dark. The cells were then washed and incubated with primary antibodies against surface markers or matched isotype control for 20 min at 4 °C. After washing, the samples were acquired using a BD FACS Canto II flow cytometer (BD Biosciences) and analyzed with the FlowJo software (Tree Star, Ashland, OR, USA).

The monoclonal antibodies used for multicolor flow cytometry were as follows: anti-CD3 (UCHT-1), anti-CD19 (HIB19), anti-CD45 (HI30), anti-CD56 (B159), anti-HLA-DR (G46-6), and anti-CD11c (Bu15), purchased from BioLegend (San Diego, CA, USA), and Fixable Viability Dye eFluor 506, purchased from eBioscience.

### Statistical analysis

Statistical analyses were performed with Prism 7 (GraphPad Software, San Diego, CA, USA). Proportions or numbers of urinary cells were compared using the Mann-Whitney *U* test. Correlation analyses were performed using Spearman’s rank correlation coefficient. Receiver operating characteristic (ROC) analysis was performed to determine the values related to renal response. Statistical significance was defined as *p* < 0.05 (*), < 0.01 (**), < 0.001 (***), or < 0.0001 (****).

## Results

### CD11c^+^ macrophages are specifically abundant in the urine of patients with proliferative LN

**Fig. 1:**
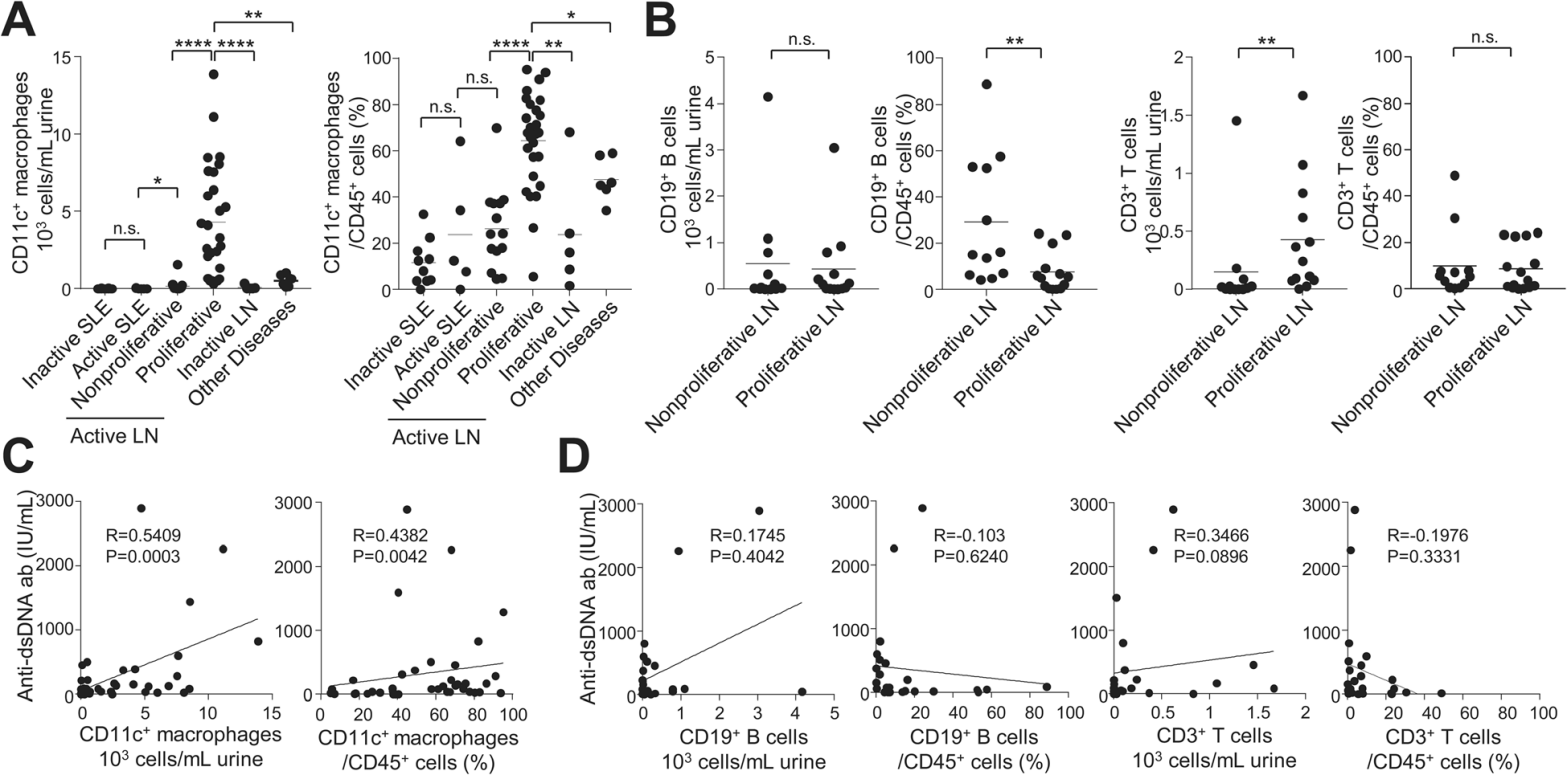
CD11c^+^ macrophages in the urine of patients with lupus nephritis (LN). **a** Numbers and proportions of urinary CD11c^+^ macrophages among CD45^+^ cells in patients with inactive systemic lupus erythematosus (SLE), active SLE, active LN (proliferative and non-proliferative), inactive LN, or other diseases with nephropathy (i.e., IgA nephropathy, ANCA-associated vasculitis) (*n* = 10, 5, 14, 27, 5, and 6, respectively). **b** The numbers and proportions of urinary CD19^+^ B cells and CD3^+^ T cells in SLE patients with non-proliferative LN or proliferative LN (*n* = 25). **c** Correlation between the numbers and proportions of CD11c^+^ macrophages in CD45^+^ cells and serum anti-dsDNA antibody titers in active LN patients (*n* = 41). **d** Correlation between serum anti-dsDNA antibody titers, and numbers and proportions of urinary CD19^+^ B or CD3^+^ T cells in LN patients (*n* = 25). *P < 0.05, **P < 0.01, ***P < 0.001, ****P < 0.0001

We analyzed CD11c^+^ macrophages (CD11c^+^HLA-DR^+^CD45^+^CD3^−^CD19^−^CD56^−^ cells) in the urine samples of patients with LN by using multicolor flow cytometry. The numbers and proportions of urinary CD11c^+^ macrophages among CD45^+^ cells were specifically and significantly higher in patients with active proliferative LN (SLEDAI 15.2 ± 5.2) than in patients with active SLE (SLEDAI 14.6 ± 5.7) without LN, those with inactive LN (SLEDAI 3.4 ± 0.9), or those with other diseases (i.e., IgA nephropathy and ANCA-associated vasculitis) (Fig. 1a). However, no significant difference was found between classes III and IV in terms of the numbers or proportions of urinary CD11c^+^ cells, indicating that the significance of urinary CD11c^+^ macrophages is not specific for a particular class within proliferative LN (Supplementary Fig. 1). Except for the numbers of CD3^+^ T cells, the numbers and proportions of urinary CD19^+^ B cells or CD3^+^ T cells among CD45^+^ cells were not significantly higher in patients with proliferative LN (Fig. 1b).

We found that the numbers and proportions of CD11c^+^ macrophages among CD45^+^ cells in the urine significantly correlated with the serum titers of the anti-dsDNA antibody, which is highly specific to LN (Fig. 1c). However, the numbers and proportions of urinary CD19^+^ B cells or CD3^+^ T cells among CD45^+^ cells were not significantly associated with the serum anti-dsDNA antibody levels (Fig. 1d). Thus, these data show that patients with active proliferative LN have a significant amount of CD11c^+^ macrophages in their urine.

### Urinary levels of CD11c^+^ macrophages correlate with the clinicopathologic features of patients with proliferative LN

**Fig. 2:**
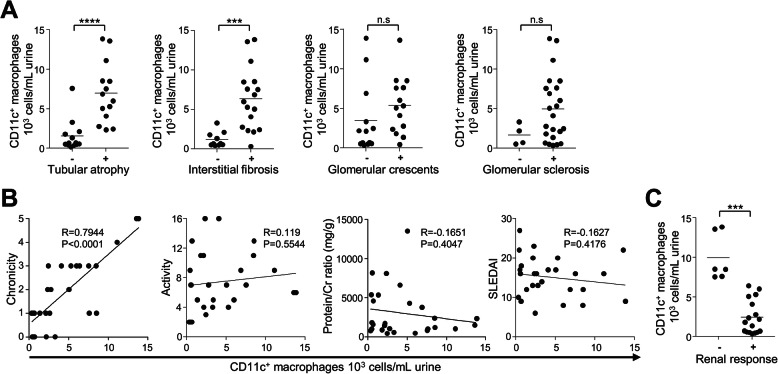
The numbers of urinary CD11c^+^ macrophages and clinicopathologic features of proliferative lupus nephritis (LN). **a** The numbers of urinary CD11c^+^ macrophages in patients with proliferative LN (*n* = 27) according to the presence of tubular atrophy, interstitial fibrosis, glomerular crescents, and glomerular sclerosis. **b** Correlation between the numbers of urinary CD11c^+^ macrophages, and chronicity, activity scores, the amounts of proteinuria, and disease activity in patients with proliferative LN (*n* = 27). **c** The numbers of urinary CD11c^+^ macrophages in patients with proliferative LN according to renal response to immunosuppressants (no [−], partial/complete [+]) (*n* = 23)

**Fig. 3:**
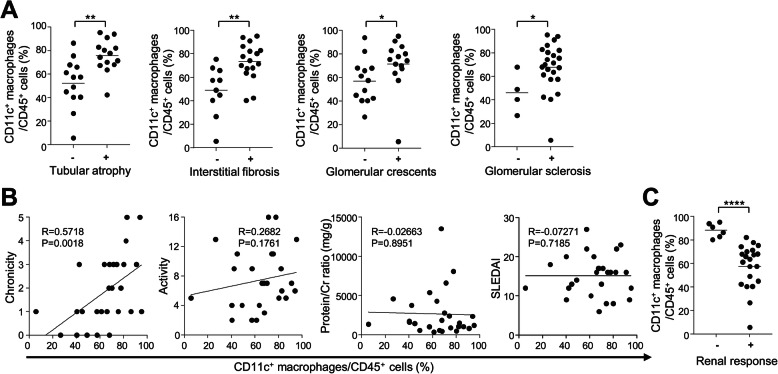
The proportions of urinary CD11c^+^ macrophages and clinicopathologic features of proliferative lupus nephritis (LN). **a** The proportions of urinary CD11c^+^ macrophages among CD45^+^ cells in patients with proliferative LN (*n* = 27) according to the presence of tubular atrophy, interstitial fibrosis, glomerular crescents, and glomerular sclerosis. **b** Correlation between the proportions of urinary CD11c^+^ macrophages, and chronicity, activity scores, the degrees of proteinuria, and disease activity in patients with proliferative LN (*n* = 27). **c** Difference in the proportions of urinary CD11c^+^ macrophages between patients with and without renal response to immunosuppressants (no [−], partial/complete [+]) (*n* = 23)

Next, we examined the histological significance of urinary CD11c^+^ macrophages in patients with active proliferative LN. We found that the numbers and proportions of CD11c^+^ macrophages among CD45^+^ cells in the urine samples, which were collected at the time of kidney biopsy, were significantly associated with the presence of tubular atrophy and interstitial fibrosis (Figs. 2a and 3a). In addition, the numbers and proportions of urinary CD11c^+^ macrophages significantly correlated with the chronicity scores, but not with the activity scores (Figs. 2b and 3b). In contrast, no correlations were found between urinary CD11c^+^ macrophages and the degree of proteinuria or lupus disease activity (SLEDAI).

**Fig. 4:**
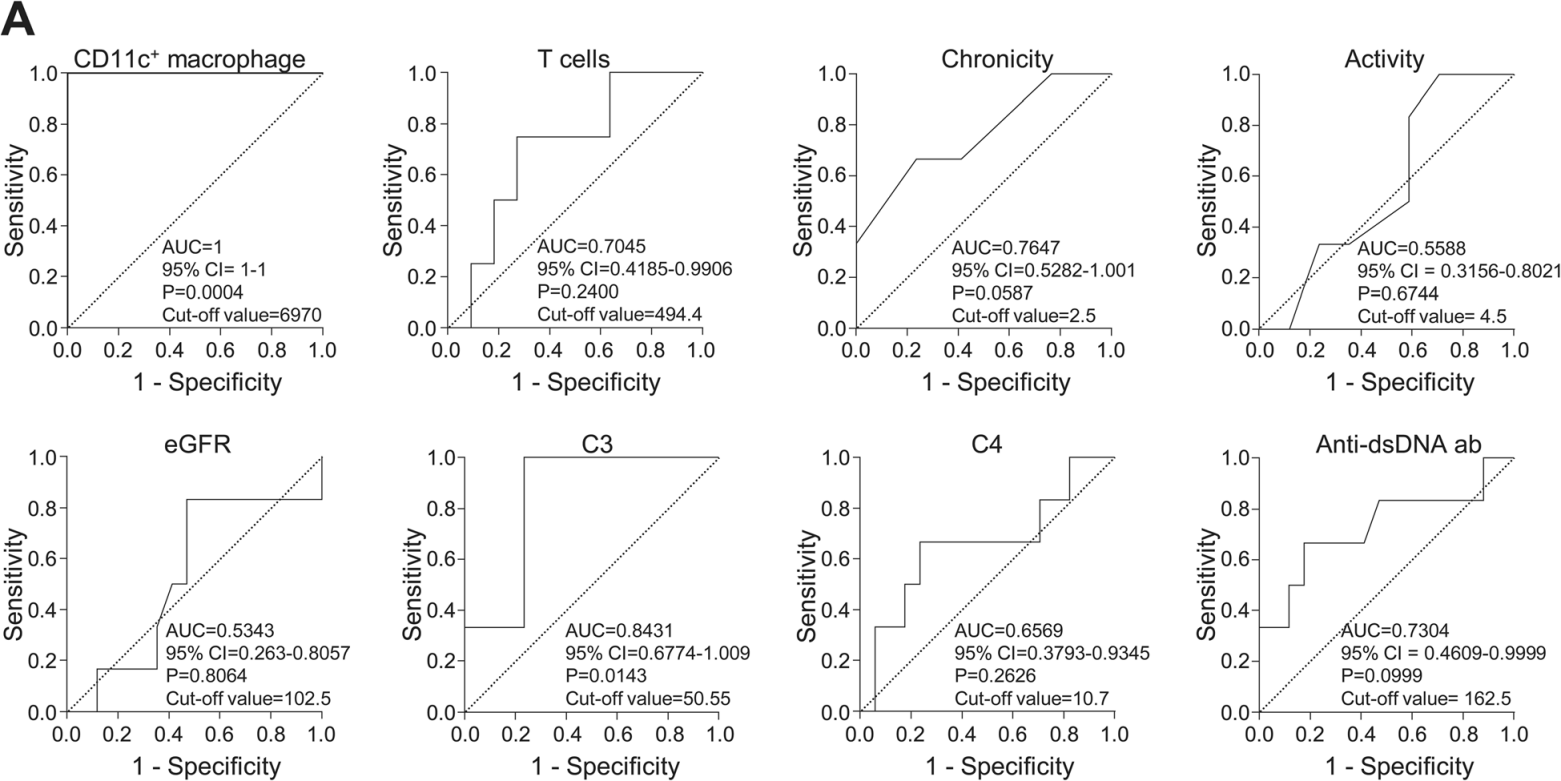
Receiver operating characteristic (ROC) curve of the factors to predictive renal response in proliferative LN. ROC curves of the predictive values of urinary CD11c^+^ macrophages, T cells, chronicity and activity scores, eGFR, C3, C4, and anti-dsDNA antibody titer values for no renal response at 6 months after treatment (*n* = 23)

Furthermore, we found that the numbers and proportions of CD11c^+^ macrophages were significantly higher in patients who did not achieve renal response at 6 months to induction treatment with immunosuppressants than in those who achieved complete or partial renal response (Figs. 2c and 3c). To determine the factors associated with treatment response in proliferative LN, ROC analyses were performed on the numbers of urinary CD11c^+^ macrophages and CD3^+^ T cells, values of chronicity, activity and eGFR, serum C3 and C4, and anti-dsDNA antibody titer (Fig. 4). The results showed that the number of urinary CD11c^+^ macrophages (AUC 1.0, 95% CI 1.0–1.0, *P* = 0.0004; cut-off value, 6970 cells/mL) was the best for predicting renal response at 6 months.

Finally, considering the results of a previous study that urinary T cells were useful for the identification of proliferative LN among SLE patients [9], we assessed the significance of urinary CD3^+^ T cells in LN. Although the numbers of urinary CD3^+^ T cells were useful for discriminating proliferative LN from total LN, we did not find significant associations between the numbers of urinary T cells and pathological features including tubular atrophy and interstitial fibrosis, the amount of proteinuria, disease activity, and renal response (Supplementary Fig. 2).

Collectively, these findings suggest that urinary levels of CD11c^+^ macrophages are closely correlated with histological and clinical features, particularly treatment responsiveness, in patients with proliferative LN.

## Discussion

In the present study, we report the significance of CD11c^+^ macrophages, whose urinary levels showed significant differences according to LN classification (proliferative LN vs. non-proliferative LN), anti-dsDNA antibody titer, and clinicopathologic features of proliferative LN such as chronicity (e.g., tubular atrophy, interstitial fibrosis, and renal response).

We addressed whether the importance of urinary CD11c^+^ macrophages on the renal response can be affected by potential confounding factors including baseline eGFR, hypertension, diabetes, and the use of steroid or cyclophosphamide. However, we were not able to adjust for potential confounding factors with urinary CD11c^+^ macrophages because the levels of CD11c^+^ macrophages were divided exclusively between the responders and nonresponders. In fact, there were no significant differences in the eGFR values and the incidence of hypertension, diabetes, and the use of steroid or cyclophosphamide between the responders and nonresponders, suggesting that the level of urinary CD11c^+^ macrophages was not significantly affected by these confounding factors (baseline eGFR, *P* = 0.995; hypertension, *P* > 0.99; diabetes, *P* = 0.261; steroid use of moderate or high dose, *P* = 0.632; cyclophosphamide, *P* > 0.99).

Previous studies have shown that infiltrating immune cells in the kidney plays an important role in the pathogenesis of LN and that T cells and monocytes/macrophages constitute the majority of the infiltrating immune cell population [16, 17]. Recently, our study on the immunological characteristics of urinary cells in patients with LN showed that CD11c^+^ macrophages were abundantly present in the urine as well as the tubulointerstitium; moreover, urinary CD11c^+^ macrophages were immunologically active, expressed proinflammatory cytokines, and interacted with tubular epithelial cells, indicating their possible pathologic role in LN [12]. Further, in the present study, we found that the amount of urinary CD11c^+^ macrophages in patients with LN had a significant correlation with the anti-dsDNA antibody titer and clinical features such as renal response to immunosuppressant treatment (Figs. 1c and 2c). Future studies are needed to elucidate the interaction between infiltrated CD11c^+^ macrophages and other immune cells such as T cells in the pathogenesis of proliferative LN.

The current pathologic classification system of the ISN/RPS is predominantly based on glomerular pathology [13, 18], but studies have shown that this classification has limited value in predicting therapy response and long-term prognosis [19–21]. This is likely due to the fact that the histological changes in the kidneys of patients with LN can include vascular as well as tubulointerstitial lesions; accordingly, the severity of tubulointerstitial lesions such as tubular atrophy and fibrosis has been shown to be more powerful than glomerular lesions in predicting poor prognosis [22, 23]. Indeed, we found that the numbers of urinary CD11c^+^ macrophages significantly differed according to the presence of tubular atrophy, interstitial fibrosis, and eventually renal response to immunosuppressive treatment (Fig. 2). These results show that the infiltrating macrophages in patients with LN may cause significant damage to the tubulointerstitium and contribute to the renal pathology and treatment response in LN.

Kidney biopsy is crucial in the diagnosis, assessment, and management of LN. However, its invasive nature results in clinical risk and difficulty of repeated collection, which calls for the identification of alternative biomarkers. Indeed, several candidate biomarkers from urinary immune cells or proteins have been previously proposed for diagnosing LN or predicting treatment response [8–10, 24–26]. However, previous works mainly focused on the value of those biomarkers for identifying patients with proliferative LN among patients with SLE. Moreover, serial measurements or a combination of several different biomarkers (i.e., biomarker panels) are necessary to offer the best discriminative ability. In the present study, we found that urinary CD11c^+^ macrophages were specifically abundant in patients with proliferative LN than in those with non-proliferative LN (Fig. 1a). The abundance of urinary CD11c^+^ macrophages well correlated with the anti-dsDNA antibody titer (Fig. 1c) and was associated with the chronicity scores (Figs. 2b and 3b) and renal response to immunosuppressants (Fig. 2c) in LN. In addition, the number of urinary CD11c^+^ cells was the strongest predictive factor for the achievement of renal response (Fig. 4). When we assessed the usefulness of urinary T cells considering the results of a previous study [9], we did not find significant associations between CD3^+^ T cells and clinicopathological features including renal response, except for the identification of proliferative LN (Supplementary Fig. 2). These findings suggest that the analysis of urinary CD11c^+^ macrophages could be a useful non-invasive biomarker as an alternative to kidney biopsy for the assessment of LN. In particular, it would be significant and useful to assess the pathological findings and predict the renal response at the time of biopsy.

## Conclusion

We demonstrated that urinary CD11c^+^ macrophages were specifically abundant in patients with proliferative LN and showed a significant correlation with the anti-dsDNA antibody titer and various clinicohistological features of LN including renal response to immunosuppressant treatment. Thus, our results suggest that urinary levels of CD11c^+^ macrophages may be a useful non-invasive indicator for the assessment of tubulointerstitial change and treatment response in patients with LN.

## Supplementary information

**Additional file 1: Supplementary Figure 1.** Comparison of the number (left) and proportion (right) of urinary CD11c^+^ macrophages between patients with Class III (*n* = 14) and Class IV (*n* = 13) LN. **Supplementary Figure 2.** The numbers of urinary CD3^+^ T cells and clinicopathologic features of proliferative lupus nephritis (LN). (a) ROC curves of the predictive values of urinary CD3^+^ T cells for proliferative LN (proliferative =14, non-proliferative = 12). (b) The numbers of urinary CD3^+^ T cells in patients with proliferative LN (n = 14) according to the presence of tubular atrophy, interstitial fibrosis, glomerular crescents, and glomerular sclerosis. (c) Correlation between the numbers of urinary CD3^+^ T cells and chronicity, activity scores, the amounts of proteinuria, and disease activity in patients with proliferative LN (n = 14). (d) The numbers of urinary CD3^+^ T cells in patients with proliferative LN according to renal response to immunosuppressants (no [−], partial/complete [+]) (*n* = 10).

## Data Availability

The datasets analyzed during the study are available from the corresponding author on reasonable request.

## Abbreviations

LN: Lupus nephritis
SLE: Systemic lupus erythematosus
SLEDAI: SLE disease activity index
ROC: Receiver operating characteristic
eGFR: Estimated glomerular filtration rate
