# Supplementary figures and images for: Clinical and histological significance of urinary CD11c+ macrophages in lupus nephritis
Source: Arthritis Res Ther. 2020 Jul 17;22:173. doi: 10.1186/s13075-020-02265-1 (PMC7368794; doi:10.1186/s13075-020-02265-1)

# Supplementary figure 1

a

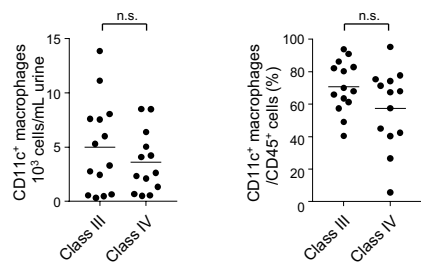

## Supplementary figure 2

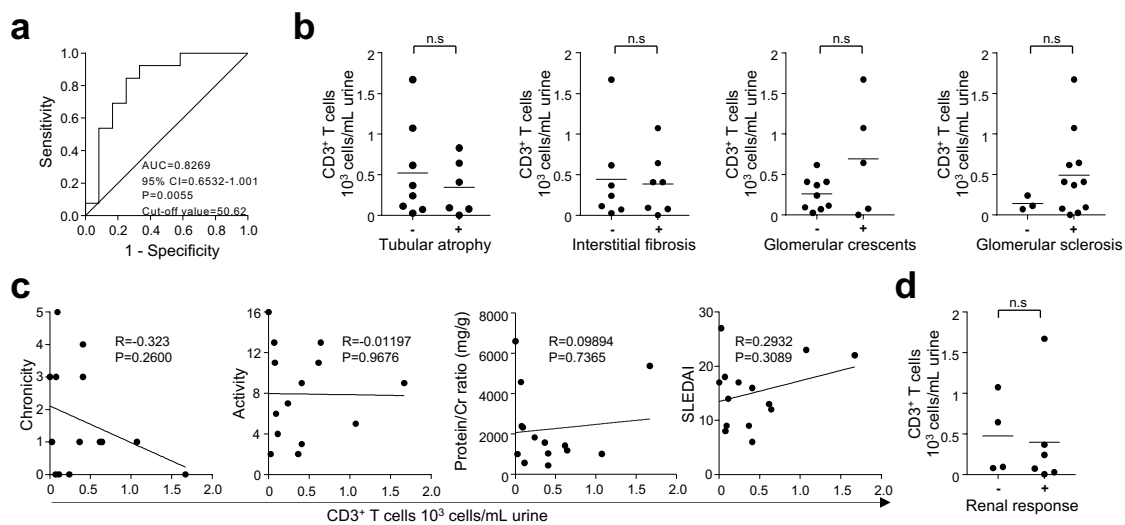

Supplement: Supplementary file 1 — Additional file 1: Supplementary Figure 1. Comparison of the number (left) and proportion (right) of urinary CD11c+ macrophages between patients with Class III (n = 14) and Class IV (n = 13) LN. Supplementary Figure 2. The numbers of urinary CD3+ T cells and clinicopathologic features of proliferative lupus nephritis (LN). (a) ROC curves of the predictive values of urinary CD3+ T cells for proliferative LN (proliferative =14, non-proliferative = 12). (b) The numbers of urinary CD3+ T cells in patients with proliferative LN (n = 14) according to the presence of tubular atrophy, interstitial fibrosis, glomerular crescents, and glomerular sclerosis. (c) Correlation between the numbers of urinary CD3+ T cells and chronicity, activity scores, the amounts of proteinuria, and disease activity in patients with proliferative LN (n = 14). (d) The numbers of urinary CD3+ T cells in patients with proliferative LN according to renal response to immunosuppressants (no [−], partial/complete [+]) (n = 10). [file 13075_2020_2265_MOESM1_ESM.pdf]
